# Area deprivation measures used in Brazil: a scoping review

**DOI:** 10.11606/S1518-8787.2018052000933

**Published:** 2018-09-03

**Authors:** Maria Yury Travassos Ichihara, Dandara Ramos, Poliana Rebouças, Flávia Jôse Oliveira, Andrêa J. F. Ferreira, Camila Teixeira, Mirjam Allik, Srinivasa Vittal Katikireddi, Mauricio L. Barreto, Alastair H Leyland, Ruth Dundas

**Affiliations:** I Fundação Oswaldo Cruz. Instituto Gonçalo Moniz. Centro de Integração de Dados e Conhecimentos para a Saúde. Salvador, BA, Brasil; II Universidade Federal da Bahia. Instituto de Saúde Coletiva. Salvador, BA, Brasil; III University of Glasgow. MRC/CSO Social and Public Health Sciences Unit. Glasgow, Scotland

**Keywords:** Poverty, Poverty Areas, Socioeconomic Factors, Social Inequity, classification, Social Indicators, Review

## Abstract

**OBJECTIVE:**

To describe and assess currently used area-based measures of deprivation in Brazil for health research, to the purpose of informing the development of a future small area deprivation index.

**METHODS:**

We searched five electronic databases and seven websites of Brazilian research institutions and governmental agencies. Inclusion criteria were: studies proposing measures of deprivation for small areas (i.e., finer geography than country-level) in Brazil, published in English, Portuguese or Spanish. After data-extraction, results were tabulated according to the area level the deprivation measure was created for and to the dimensions of deprivation or poverty included in the measures. A narrative synthesis approach was used to summarize the measures available, highlighting their utility for public health research.

**RESULTS:**

A total of 7,199 records were retrieved, 126 full-text articles were assessed after inclusion criteria and a final list of 30 articles was selected. No small-area deprivation measures that have been applied to the whole of Brazil were found. Existing measures were mainly used to study infectious and parasitic diseases. Few studies used the measures to assess inequalities in mortality and no studies used the deprivation measure to evaluate the impact of social programs.

**CONCLUSIONS:**

No up-to-date small area-based deprivation measure in Brazil covers the whole country. There is a need to develop such an index for Brazil to measure and monitor inequalities in health and mortality, particularly to assess progress in Brazil against the Sustainable Development Goal targets for different health outcomes, showing progress by socioeconomic groups.

## INTRODUCTION

Brazil is a country with high rates of inequality, with the benefits of economic growth having been distributed inequitably^1^ ^,^ ^2^. Although overall poverty rates have declined and global measures of socioeconomic conditions, such as the Human Development Index (HDI), have been showing consistent improvements in recent decades, internal disparities remain substantial. These disparities exist across both urban and rural contexts and have persisted during urbanization^3^ ^-^ ^5^.

Given that socioeconomic status is one of the most consistent determinants of health^6^ ^,^ ^7^, identifying those sub-populations that are still living in poverty, and being left behind despite economic growth, is an important task. Such identification allows the monitoring of health inequalities, the understanding of their causes and facilitates the evaluation of the impact of social programs on health inequalities – all of which helps achieve health equity.

To achieve this goal, several studies have sought to calculate poverty and deprivation indexes. In this review, our aim is to establish the current state of scientific knowledge about measures of deprivation for the Brazilian population, through a scoping review of scientific databases and grey literature.

### Individual Socioeconomic Position and Area Deprivation

Area-based deprivation indexes have been used in the UK^8^ ^,^ ^9^ for over 30 years, and they are also available for many other countries, such as New Zealand^10^, Canada^11^ ^,^ ^12^, France^13^ and Germany^14^. These deprivation indices are important to understand social inequalities in general and health inequalities. They describe the socioeconomic characteristics of an area that comprises individuals and not the individuals themselves. This feature demonstrates the utility of the deprivation index as it can be used in situations where the individual-level variables of socioeconomic position are not available or are poorly recorded for certain subgroups (e.g., occupational social class of elderly people who have not worked for a number of years)^9^. Area deprivation measures are also used when individual-level measures are available; individual socioeconomic position and area deprivation can both have an effect on outcomes, independent of each other^15^. Area-based deprivation indicators can be linked to routinely collected administrative data, such as mortality and hospitalizations, where there is typically no information about the socioeconomic position of the individual. These can then be used to describe the extent of inequalities in the health outcome. Area-based deprivation indices are also used by policymakers to focus policies, funding or interventions on those most in need.

### Individual Socioeconomic Position and Area Deprivation in Brazil

While we prefer the term deprivation to describe area-based measures of socioeconomic position, the term poverty is used extensively in the Brazilian literature. The use of income-based indicators of poverty are still dominant in the Brazilian literature and in the official government measures, but there is no consensus on the optimum measure^16^. The *Bolsa Família* eligibility criteria defines as poor or extremely poor all families with *per capita* income below 170 Reais and 85 Reais, respectively^17^, while the Brazilian Institute of Geography and Statistics (IBGE) defines *per capita* income of one-quarter of the minimum wage (234.25 Reais) as extreme poverty and one-half of the minimum wage (468.50) as poverty, based on the average living cost for the Brazilian population^18^.

The main source for the calculation of such indicators for Brazil has been the Brazilian National Household Sample Survey (PNAD), conducted annually by IBGE since 1981. PNAD investigates several characteristics of the population such as household composition, education, labor, income and fertility. However, the PNAD collects data only about the families’ expenditures on basic needs, not allowing the study of other forms of deprivation beyond income, such as the different levels of multidimensional poverty^19^.

The study of multidimensional measures of poverty and deprivation for the Brazilian population has mainly been conducted by the Brazilian Institute of Geography and Statistics^20^ and the Ministry of Social Development, who released a series of technical reports on how to calculate and apply such measures using census data or social registry data^21^ ^-^ ^23^. Barros et al. have also developed a multidimensional poverty index by family, called IDF (Family Development Index)^24^, and Kageyama and Hoffman have proposed the study of the spatial distribution of poverty in the country, based on the Basic Human Needs approach^25^.

The purpose of this review is to inform the development of a small-area deprivation index for Brazil. Therefore, the aim is to review the literature to describe currently used area-based measures of socioeconomic inequalities in Brazil and assess the scope for using area-level deprivation measures for health research in Brazil. Specific research questions (RQ) are

RQ1 – What is the population coverage of the measure?

RQ2 – What area level is the measure operationalized at?

RQ3 – What dimensions of poverty are included in the measure?

RQ4 – What years and data sources are used in the measure?

## METHODS

To describe the existing evidence on deprivation indexes for the Brazilian population, we performed a scoping review^26^ ^-^ ^28^. Sources were considered for inclusion in the scoping review when they were consistent with the Population, Concept and Context (PCC). In this review, the population was areas in Brazil, the concept was measures of multiple deprivations and the context was small areas (i.e., finer geography than country-level) in Brazil.

### Databases and Search Strategies

We searched five electronic databases (SCOPUS, PubMed, Web of Science, SocINDEX, Latin American and Caribbean Health Sciences Literature [LILACS]) on 1st September 2017, with no date restrictions for searches. Additional records and grey literature were retrieved through targeted searches (search terms: poverty/*pobreza*, deprivation/*privação*, small areas/*pequenas áreas*, census/*censo*) on the websites of Brazilian research institutions and governmental agencies, such as Getúlio Vargas Foundation (FGV), Brazilian Institute of Advanced Economic Research (IPEA), Pan-American Health Organization (OPAS/OMS), Ministry of Social Development (MDS), Brazilian Institute of Geography and Statistics (IBGE), World Bank and Repositório ARCA-FIOCRUZ. Potentially eligible references cited by included articles were also screened for inclusion. The predefined search strategies were built in an adaptive form to each search platform. Search strings one to four were used in SCOPUS, PubMed, SocINDEX, Web of Science and LILACS, and search string five was used only on PubMed.

1. (Poverty AND (measurement OR index) AND Brazil)
2. ((Poverty OR Poor) AND (measurement OR index) AND Brazil)
3. ((“Poverty measurement”) OR (“poverty index”) AND Brazil)
4. ((“Poverty measurement”) OR (“poverty index”) OR (“multidimensional poverty”) AND Brazil)
5. ((“Poverty”[Mesh] OR poverty[TIAB]) AND (Brazil”[Mesh] OR Brazil[TIAB]) AND (MEASUREMENT OR INDEX))

### Eligibility Criteria

We reviewed abstracts and full texts of retrieved articles according to the following inclusion criteria: 1) included Brazil (or parts of Brazil); 2) were defined at the area level (e.g., not individual socioeconomic status) and; 3) reported a measure of social stratification (e.g., area-based poverty or deprivation or inequality). The exclusion criteria were: I) published in a language other than English, Portuguese or Spanish; and II) full text not available. There was no restriction by publication year. There were no restrictions on the sources of information that could contribute to the scoping review; all types of existing literature from the databases searched were included.

### Data

All relevant data were extracted from the full texts by two reviewers, DR and RD. The data extracted related to the aim of the scoping review to identify the measures that have been used to report area-based deprivation in Brazil. We extracted data on study characteristics (publication year, journal and authors), name of the deprivation measure, area level (e.g., municipalities, census tracts), geographical coverage (e.g., the proportion of the Brazilian population that has a valid measure available), variables used to calculate the index and whether it was used to report on a health outcome.

### Analysis

We did not critically appraise the included studies, as is common in scoping reviews^26^ ^,^ ^29^ ^,^ ^30^. Data were managed with the assistance of Endnote X3 for reference management and Microsoft® Excel 2010 for data extraction and summarising findings. A narrative summary approach to data analysis was undertaken^31^. Results were tabulated according to the area-level that the deprivation measure was created for and dimensions of deprivation or poverty included in the measures. We summarised the different deprivation measures available, highlighting their utility for applying to public health research. In particular, we summarised information according to the population coverage of the measure, the area level used, dimensions of deprivation incorporated, and the data sources used.

## RESULTS

A total of 7,199 records were retrieved. Of these, 3,808 were duplicates, and 3,265 were excluded after title screening, leaving a total of 126 full-text articles for assessment. Reasons for exclusion during full-text review appear in Figure, with most of the studies excluded because they did not focus on area-level measurements (inclusion criteria #2, n = 69), extracting information only from individual or household level data. After all the screening stages and inclusion/exclusion criteria, a final list of 30 articles was selected.

**Figure:**
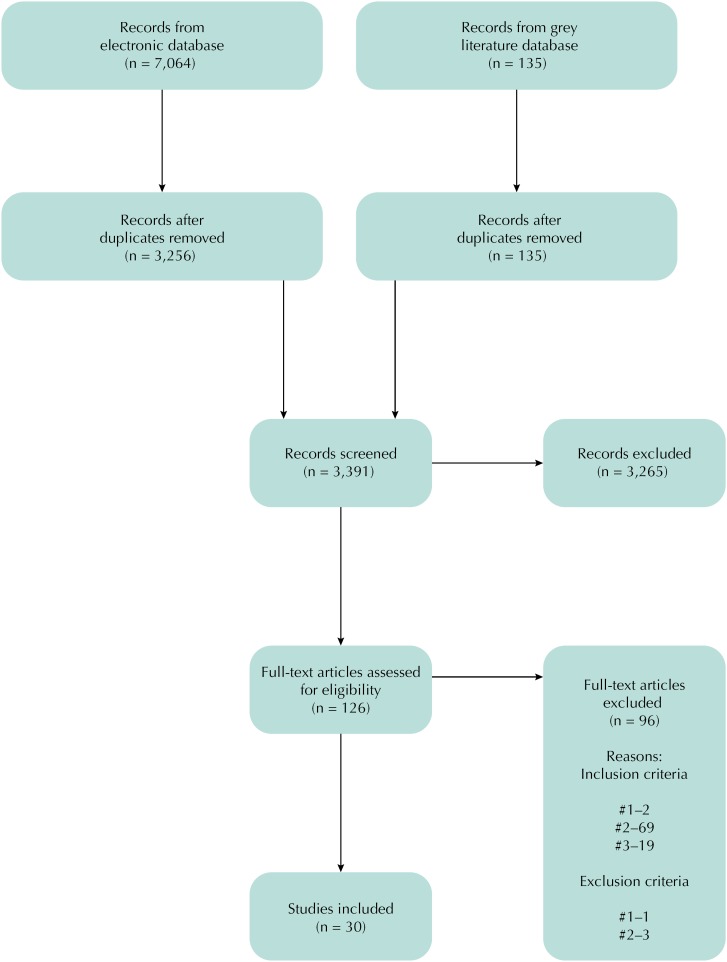
PRISMA flow diagram for the scoping review process.

As shown in Table, 15 studies were published from 2000–2010 and 15 from 2011–2017. There were four main area-levels that were considered in the papers: municipalities (with an average population of 37,000)^32^ ^-^ ^35^; census tracts (average population 660)^7^ ^,^ ^36^ ^-^ ^38^; districts (average population 20,000)^36^ ^,^ ^39^ ^-^ ^42^; and neighborhoods^43^ ^-^ ^46^. Six papers considered other area levels^7^ ^,^ ^47^ ^-^ ^51^. The majority of the papers examined health outcomes by area-level deprivation; only six papers did not have a health outcome. These were mainly in the field of infectious/parasitic diseases. Two papers reported all-cause mortality and three further studies examined mortality from homicide specifically.

**Table:** Characteristics of the 30 studies of deprivation indexes.

Characteristics		n (%)
Publication year		
	2000-2010	15 (50)
	2011-2017	15 (50)
Area level²		
	Municipalities	9 (30)
	Census tracts	6 (20)
	Districts	5 (17)
	Neighborhoods	4 (13)
	Others	6 (20)
Health outcomes²		
	Infant/Childhood mortality	6 (20)
	Homicides	4 (13)
	Teenage pregnancy	2 (7)
	Infectious/Parasitic diseases	10 (33)
	Mortality	2 (7)
	Oral health	3 (10)
	Others	2 (7)
	No study of health outcomes	6 (20)

* This categorization refers to the terms used by the authors on the reviewed studies. The sum adds to more than 30 because some studies created measures for more than one area-level, simultaneously.

The Box groups the papers by the area level used and displays the dimensions of deprivation considered by each of the papers. There is a noticeable growth in the scope of the studies over time as shown by the number of dimensions used. This is a possible result of the improved availability of data and the modernization of the Brazilian information systems. Key themes are presented below in relation to each research question.

**Box:** Dimensions and indicators of deprivation used in the measures reported by the 30 reviewed studies, ordered by area level.

Author (year)	Dimension	Indicators
		Municipality
Barata et al.^38^ (2000)	Income	Average monthly income of household heads; Gini coeficient; Income concentration index (ratio between the income of the 90th percentile/income of the 20th; percentile).
Bezerra Filho et al.^33^ (2007)	Income Sanitary conditions Housing conditions Education	Proportion of financial resources to health in the municipality; proportion of household heads according to income in minimum wages; proportion of children from 10 to 14 years old working; proportion of the value of vegetal production compared to the total of the state, proportion of rural, industrial, commercial energy use on the total of the municipality, *per capita* gross domestic product; intensity of poverty (line of R$37.75) and IDHM Income; Proportion of the population with access to a public supply of water, sewage facilities, trash collection; access to power supply; Proportion of small houses (two or less rooms); Proportion of people living in a house with telephone; Proportion of illiterate children from seven to 14 years old, proportion of children from seven to 14 not in school, literacy rate among adults, average number of schooling years of people who were 25 or older; proportion of literate women and proportion of women with < 8 years of schooling; and component for education of the Human Development Index (IDH).
Drachler et al.^34^ (2014)	Income Sanitary conditions Education Other	Proportion of households with monthly income below 1/2 minimum wage; Proportion of households not connected to the water distribution system; proportion of households with no connection to the sewage system; proportion of households without waste or garbage collection; Proportion of illiteracy among people above 15 year of age; Demographic density.
Ottoneli et al.^35^ (2014)	Income Sanitary conditions Housing conditions Education Other	Average income of the households; proportion of households with income below 1/2 of the minimum wage; Proportion of households with access to the sewage system; proportion of households with access to the water distribution system; proportion of households with access to garbage removal or collection; proportion of households with a bathroom; Proportion of households with electricity; Proportion of households with density of dwellers above 2; Proportion of households with refrigerators; literacy rate of people above five years of age; proportion of illiterate people between five and 14 years of age; proportion of illiterate people above 15 years of age; proportion of households where the head of the household was illiterate; proportion of people above 10 years of age with no schooling or below 5th grade; proportion of people above 10 years of age who completed high school; infant mortality; child mortality (under-5); proportion of children and adolescents (10–17 years) who had children.
Costa et al.^52^ (2016)	Income Housing conditions Education Other	Percentage of poor people; Percentage of households built with durable material; Illiterates aged 15 or over; Infant mortality per 100 thousand live births.
Vieira et al. (2017)^63^	Income Sanitary conditions Housing conditions Education Working/ Employment	Household income [R$154.00/person living at home (in 2010)]; 1/2 minimum wage *per capita* (in 2000); Water supply (general network with internal conduits, general network without internal conduit, well or spring with internal piping, well or spring without internal piping, other way); sanitary facilities (no or yes access to toilet facilities); type of drainage system for toilets (general network, septic tank, rudimentary fossa, another sewer); waste destination (collected by cleaning service, collected in cleaning service bucket, burned, buried, played in wasteland, played in river, lake or sea; another destination); Number of bathrooms at home (no bathroom or at least one); Condition of occupation of the property (owned, leased, given by an employer, assigned otherwise, another condition); electricity (yes or no at the household); goods (cable TV, refrigerator and washing machine); Literacy and non literacy; years of education (no education or less than three years of study; from four to seven years; from eight to 10 years; from 11 to 14 years; 15 or more years of study); Employment (no or yes – access to a job).
Castro et al.^59^ (2016)	Income Working/ Employment Other	Gini index (income inequality of household heads); average household income *per capita*; proportion of the poor population (those with *per capita* income below half the minimum wage); proportion of poor children; Unemployment rate of the population aged 18 years or older; Municipal Human Development Index (IDHM).
Cortellazzi et al.^53^ (2014)	Income Housing conditions Education Other	*Per capita* income; Population density; Educational level of the adult population; educational flow of the younger population; Municipal Human Development Index (IDHM).
Junior et al.^60^ (2014)	Income Sanitary conditions Education	*Per capita* income (the mean declared monthly income in multiples of the minimum wage); Basic sanitation (percentage of households connected to public sewage or drainage system); refuse collection (percentage of households with public refuse collection); public water supply (percentage of households connected to a public water supply); Literacy rate (the percentage of the population able to read and write).
		**Census tracts**
Szwarcwald et al.^55^ (2002)	Income	Index of heterogeneity of poverty concentration among sub-areas – household head monthly income.
Bonfim et al.^36^ (2009)	Income Sanitary conditions Housing conditions Education	Proportion of heads of households with an income of half to one minimum monthly salary; Proportion of households with water supply from wells or springs only in the yard of the property, not piped, and other forms of water supply; proportion of households with sewage disposal into a rudimentary cesspit, ditch or gutter, or into a river, a lake or the sea, and without a bathroom; proportion of households in which garbage is burned, buried, dumped on vacant land or in rivers, or other destinations; proportion of households that are not owner-occupiers or living in rented or assigned property; proportion of households consisting of 10 people or more; Proportion of heads of households with not more than one year of schooling.
Bonfim et al.^37^ (2011)	Income Sanitary conditions Housing conditions Education	Proportion of heads of households with an income between 0.5 and 1.0 minimum wages; Proportion of households with inadequate water supply; proportion of households with inadequate sewage sanitary; proportion of households with inadequate solid waste collection; proportion of households with ten or more residents; proportion of rented households; proportion of heads of households with < 1 year of schooling; proportion of the population aged between 10 and 14 years with no schooling.
Hino et al.^38^ (2011)	Income Housing conditions Education	Proportion of household heads with income below two minimum wage; Household density; Proportion of illiterate people between ages 10–14; proportion household heads with less than three years of formal education.
Dias et al.^61^ (2016)	Income Sanitary conditions Housing conditions Education Other	Income (*per capita* income up to 1/2 minimum wage, responsible people's average income); Sanitation (inadequate water supply, inadequate sanitary sewage, inadequate garbage collection); Housing (residents per household); Education (illiterate population); Social (black and indigenous people's percentage).
Santos et al.^62^ (2007)	Income Housing conditions Education	Average income (in Reais) of people responsible for each household; average income (in Reais) of the women responsible for each household; Rate of households with more than five inhabitants; Average years of schooling of people responsible for each household; average years of schooling of the women responsible for each household; rate of illiterate people over five years of age; rate of illiterate women over five years of age.
		**Districts**
Lima et al.^41^ (2005)	Income Education Other	*Per capita* family income, inequality index, Gini index, average income of the head of the family, poverty index; Rate of illiteracy; Demographic density.
D’Ambrosio et al.^40^ (2008)	Housing conditions Education Working Employment Other	Lives in a favela; his or her dwelling is “improvised”; his or her dwelling is one-room type; his or her dwelling is overcrowded; Does not have (or has not had) access to formal education; Uemployed; domestic paid worker; Lives in a rural area; lives in a polluted area; lives in a place not served by good urban services; does not have access to a minimum standard of consumption.
Bonfim et al.^36^ (2009)	Income Sanitary conditions Housing conditions Education	Proportion of heads of households with an income of half to one minimum monthly salary; Proportion of households with water supply from wells or springs only in the yard of the property, not piped, and other forms of water supply; proportion of households with sewage disposal into a rudimentary cesspit, ditch or gutter, or into a river, a lake or the sea, and without a bathroom; Proportion of households in which garbage is burned, buried, dumped on vacant land or in rivers or other destinations; proportion of households that are not owner-occupiers or living in rented or assigned property; Proportion of households with 10 people or more; Proportion of heads of households with not more than one year of schooling.
Oliveira et al.^42^ (2009)	Income Sanitary conditions Education	Income; Sanitary household conditions; water quality; Schooling.
Antunes et al.^39^ (2002)	Income Sanitary conditions Housing conditions Working/Employment	Household income; Gini coefficient for income inequality; Water supply; Household overcrowding; Unemployment rate.
		**Neighborhood**
Duarte et al.^45^ (2006)	Income Sanitary conditions Education Other	Income level of the household head; Proportion of households with access to the sewage and water distribution system; proportion of households with access to garbage removal; Coverage of daycare centers for children below four years of age; school coverage for children with ages between 4–6; Concentration of female illiteracy; Coverage of basic health unit per 5.000 inhabitants; Concentration of women as household heads; Concentration of precarious households.
Lopes et al.^46^ (2015)	Income Sanitary conditions Housing conditions Education	Proportion of people below the poverty line; Proportion of household in adequate living conditions; proportion of households with a bathroom; Proportion of households with more than seven people; Proportion of illiterate people.
De Holanda et al.^43^ (2015)	Housing conditions Education Other	Proportion of homes in the poverty range; Illiteracy rate; illiterate women responsible for the home; Pregnant women without prenatal care.
De Sousa et al.^44^ (2014)	Income Sanitary conditions Education Other	Average nominal income; *per capita* income; proportion of people with high incomes; Proportion of heads of households under the poverty line and below it; proportion of poor households; Proportion of households with water supply; proportion of households with garbage collection; Proportion of heads of households in relation to years of education; average years of education; proportion of people with early and late literacy; Human Development Index per neighborhood; Proportion of youth and longevity.
		Other areas
Szwarcwald et al.^7^ (2000)	Income	Proportion of household heads who earned less than one “minimum wage” per month.
Rocha et al.^49^ (2000)	Other	Food expenses (food basket).
Teixeira et al.^51^ (2002)	Other	No Information.
Barros et al. ^54^ (2003)	Income Sanitary conditions Housing conditions Education Working Employment Other	Income; familiar income; Access to water supply, sewage system electricity, garbage collection and goods; Type of residence; Schooling by age; Professional qualification; number of children out of school in the family; Number of people active for work and occupied; Number of live births; Presence of children, adolescents, young people and older adults in the family; presence of the mother in the family; presence of mother who has had stillbirth or a child who has died; Density of residents.
Barbieri et al.^48^ (2015)	Income Sanitary conditions Education Working/ Employment Other	Proportion variation in GRP; Proportion of households with proper sanitation; Expected education; Proportion variation in employment; Average prevalence of dengue; average prevalence of respiratory diseases; average prevalence of infectious diseases; proportion variation in family consumption; government transfer support ratio; proportion of municipalities with risk management plan; total dependency ratio.
Antunes et al.^47^ (2004)	Income Education Other	Income; insufficient Income; Illiteracy rate; Human Development Index; Child Development Index.
Souza et al.^50^ (2012)	Income Sanitary conditions Housing conditions Education Other	Income (proportion of heads of family in permanent private households with a mean monthly income ≤ 2 minimum salaries); Sanitation (percentage of households with an internal water supply connected to the mains); Slum (percentage of households within subnormal clusters); agglomeration (ratio of inhabitants per room); Education (proportion of literate individuals aged 10–14 years in the population); Living conditions index (LCI).

### Population Coverage of the Measure

Most papers only created the area-level measure of deprivation for specific areas (e.g., states, regions, cities) within Brazil and not for the entire country. Three papers created their measure for the whole of Brazil; Costa et al. and Cortellazzi et al. used the municipality area-level^52^ ^,^ ^53^ and Barros et al. used the Regions and States^54^; none of them was at the census tract level. Costa et al. and Cortellazzi et al. used data from 2010 Census^52^ ^,^ ^53^, Barros et al. used data from 1991 and 2000 Census^54^.

### Area Level of the Measure

Four main area levels were considered in the papers: municipalities; census tracts; districts; and neighborhoods. Six papers considered other areas, as follows: towns (no information provided regarding average population size), information zones (average population 2,190,849), microregions (average population 19,595,309), administrative regions (average population 5,000,000) and metropolitan regions (no information provided regarding average population size).

### Dimensions of Deprivation Included in the Measure

Many papers used the Human Development Index of Municipalities (IDHM) developed by the United Nations Development Programme (UNDP). This is created using three dimensions of human development – longevity, education and income. The IDHM was used as a standalone measure and as a dimension of other measures.

Nearly all papers included income as a dimension of deprivation. Only four did not include income. Income was assessed in different ways; these include average nominal income; *per capita* income, proportion of people with high incomes, homes in the poverty range, proportion of heads of family in permanent private households with a mean monthly income ≤ 2 minimum salaries. The 21 studies included education as an indicator of deprivation; the main measures were illiteracy and years of education. Sanitary and housing conditions were also important dimensions of deprivation and were included in most papers. A few studies used employment as an indicator.

Other indicators included food habits and costs, access to health services and a general measure of inequality such as the concentration index or Gini coefficient. It should be noted that general measures of inequality do not measure deprivation, but measure heterogeneity of poverty concentration in a geographic location^55^.

### Years of Data Collection for the Dimensions of Deprivation

Most data to create the area level deprivation indices came from the Brazil Censuses of 1990 (six papers), 2000 (eight papers) and 2010 (nine papers).

From the two papers that reported all-cause mortality and three further studies that examined mortality from homicide specifically, none of these covered the whole population of Brazil and none was at the census tract level. Two were at the district level, and the others were at the neighborhood, municipality and administrative region. These were all carried out using 1991 or 2000 Census data; none used the 2010 Census.

## DISCUSSION

This review summarizes the efforts made in Brazil to create area-based deprivation indices. While we identified a few studies that developed deprivation measures applied to the whole of Brazil, none of them was based on small areas (census tracts). Three studies did cover the entire country but the “small areas” used were municipalities with an average population of 37,000. We found limited deprivation measures using the census tract area level and limited deprivation measures using the most recent 2010 Census. In addition, the small area deprivation measures were mainly used to describe inequalities in infectious and parasitic diseases. Few studies used the deprivation measure to assess inequalities in mortality and no studies used the deprivation measure to evaluate the impact of social programs designed for poor populations (e.g., *Bolsa Família, Minha Casa Minha Vida*).

This scoping review has several strengths. A comprehensive search strategy was used; we searched five electronic databases, including a key repository for LILACS. A standard data extraction form was used for each paper and report included in the scoping review. This means our data should be as robust and standardized as possible. There are some limitations to the scoping review. This review needed to be as comprehensive as possible, therefore we included all studies, regardless of the quality. A risk of bias tool was not used to assess studies for quality or inclusion; all results were given the same weight and importance. It is usual to use more than one reviewer to check a proportion of the screened results and data extraction. Results were only screened by a single person (either DR, PR or RD). DR checked a minimal number of papers (n = 14), assigned to RD to screen, where the title was in English but the abstract was only available in Portuguese, but this was not a systematic approach. However, we did not identify any further articles for inclusion based on scrutinizing the references of included articles, suggesting that we have identified the existing deprivation measures within Brazil. Similarly, only one person extracted the data from each included paper; there was no cross-checking of the data extraction. The use of single persons to screen and extract the data may have introduced bias, in that some studies may have been inadvertently excluded or the wrong information extracted. However, the information to be extracted was basic information about the deprivation measure and the data used to create it. As such, we felt we did not need a second data extractor.

### Advantages and Disadvantages of Using Area-Based Deprivation Measures

There are advantages and disadvantages of using area-level deprivation measures compared to individual measures of socioeconomic position. The appeal of area-level measures is that they are available for the entire population. Children and adolescents can have an area-based deprivation assigned to their area of residence, whereas these important sub-groups are usually excluded from individual-level analyses as they do not have, for example, an individual income or employment category. A further appeal of area-based measures, if they are at a suitably small area, is that policymakers can use them to target specific areas of deprivation. This targeting aims to ensure that those that are in the most need are able to access the services and policies required.

Potential disadvantages include the fact that, since it is an area measure and not a measure about the individuals, it might not reflect the circumstances of all individuals within an area; not all areas marked as deprived will contain only poor individuals and not all poor individuals live in deprived areas^56^. This will be less of an issue the smaller the area, as areas become more homogenous in terms of deprivation. Current research using area-based measures of deprivation in Brazil are focused at the municipality level which has an average population size of 37,000, but some municipalities are large cities, such as São Paulo or Salvador with populations of 12 and 2.9 million, respectively. These areas will certainly be extremely heterogeneous in terms of deprivation; the variation in deprivation within a municipality may be as large as the variation in deprivation between different municipalities. The use of smaller areas in terms of population size will mitigate the effects of heterogeneous areas. A further disadvantage is that areas of extreme deprivation may become stigmatized and therefore the individuals within these areas may experience disadvantage as a consequence of using deprivation measures^57^.

### Utility of an Area-Based Deprivation Index for Brazil

Using IBGE Demographic Census Data, it will be possible to develop a deprivation index based on small areas (census tracts) and, in the Brazilian context, a deprivation index can be useful for a variety of reasons. These include: a) a means to identify subpopulations that are still living in poverty, regardless of the country's trends of economic growth; b) targeting groups that are at-risk for poverty-related diseases (e.g., diarrheal infections, malnutrition); c) evaluating the impact of social programs designed for poor populations (e.g., *Bolsa Família, Minha Casa Minha Vida*); and d) through data linkage with other health information systems, exploring the role of deprivation as a causal or associated factor for many health outcomes.

The WHO Commission on Social Determinants of Health (2008) stated that measuring and monitoring inequalities in health was key to understand how to reduce inequalities^58^. Despite a history of social protection policies, 27% of the population was still in poverty in 2015, according to the criteria of the IBGE (household *per capita* income < half the minimum wage). However, using the criteria of the Ministry of Social Development, the entity responsible for social policies, such as *Bolsa Família*, this percentage is estimated to be of 40% (household *per capita* income < 140 Brazilian Reais). There is limited knowledge of where these people live and the nature of the area they live in. Area-based deprivation indicators have been in use in the UK and other countries for over 30 years. There is a need to develop a similar small-area deprivation index for Brazil that could be used to measure and monitor inequalities in health and mortality, in particular, to measure the progress in Brazil against the Sustainable Development Goals targets for different health outcomes, showing progress within socioeconomic groups.
